# Validation of the DIVAS-Q (Day-to-Day Impact of Vulvovaginal Atrophic Symptoms Questionnaire): a Portuguese patient-reported outcome measure

**DOI:** 10.1186/s41687-026-01172-4

**Published:** 2026-08-14

**Authors:** Andreia Antunes, Patrício Costa, Ana Alves, Alexandra Colaço, Maria Guarino, Cristina Nogueira-Silva

**Affiliations:** 1 Life and Health Sciences Research Institute (ICVS), University of Minho, Braga, Portugal; 2 ciTechCare -Center for Innovative Care and Health Technology, Instituto Politécnico de Leiria, Leiria, Portugal; 3 Center for Psychology at University of Porto (CPUP), University of Porto, Porto, Portugal; 4 Gynaecology and Obstetrics Service, Local Health Unit of the Leiria Region, Leiria, Portugal; 5 Personalized Health Care Unit of Sete Rios, Santa Maria Local Health Unit, Lisbon, Portugal; 6 Department of Obstetrics and Gynaecology, Braga Local Health Unit, Braga, Portugal; 7 ICVS/3B’s - PT Government Associate Laboratory, Braga/Guimarães, 4710-057, Portugal

**Keywords:** Vulvovaginal atrophy, Quality of Life, Patient-reported outcomes, Questionnaire validation, Female sexual function

## Abstract

**Introduction:**

Portuguese is the fifth most spoken language worldwide. However, no validated patient-reported outcome measure (PROM) is available to assess the impact of vulvovaginal atrophy (VVA) symptoms in Portuguese-speaking women. This study aimed to culturally adapt and psychometrically validate the European Portuguese version of the Day-to-Day Impact of Vaginal Ageing questionnaire (DIVA), herein renamed DIVAS-Q (Day-to-Day Impact of Vulvovaginal Atrophy Symptoms Questionnaire), to reflect vulvovaginal atrophy Symptoms across different age groups. Also, deliberately broadened the conceptual scope for symptomatic adult women across reproductive stages in Portugal.

**Design and methods:**

A multicentre observational study was conducted in Portugal, including pre- and post-menopausal women reporting at least one symptom of vulvovaginal atrophy. Participants were recruited from four clinical settings (public and private hospitals, private practice, and primary health care). Translation followed a standardised, multistep forward-backwards cultural adaptation protocol. Psychometric evaluation comprised descriptive analysis, confirmatory factor analysis (CFA) with WLSMV/DWLS estimation for ordinal indicators, internal consistency (Cronbach’s alpha and McDonald’s omega), item-total correlations, test-retest reliability using intraclass correlation coefficients (ICCs), convergent and discriminant validity, and known-groups validity based on the Vulvovaginal Symptom Questionnaire (VSQ) symptom-burden groups.

**Results:**

A total of 291 women completed the short version (19 items), and 243 sexually active women completed the long version (23 items) of the DIVAS-Q. CFA with WLSMV/DWLS estimation supported the original four-factor structure (daily activities, emotional well-being, sexual function, and self-concept/body image) for both versions, with good model fit (scaled CFI/TLI = 0.981/0.978 and RMSEA = 0.027 for the short version; scaled CFI/TLI = 0.973/0.970 and RMSEA = 0.033 for the long version; SRMR = 0.035-0.040). Internal consistency was excellent across all domains (Cronbach’s alpha = 0.856-0.960), with strong item-total correlations and average variance extracted values above recommended thresholds. Test-retest reliability was excellent (ICC = 0.982-0.994; 95% CIs consistently above 0.95). Known-groups validity was supported by significantly higher DIVAS-Q scores among women with higher VSQ symptom burden, with large effect sizes across domains.

**Conclusions:**

The European Portuguese DIVAS-Q demonstrated good psychometric properties for assessing the day-to-day impact of vulvovaginal atrophy-related symptoms among symptomatic adult women in Portugal. The findings support its use in clinical and research contexts in Portugal, but further studies are needed before extending its use to other Portuguese-speaking populations or using it to monitor clinically meaningful change over time.

**Supplementary Information:**

The online version contains supplementary material available at 10.1186/s41687-026-01172-4.

## Introduction

Age-related hormonal changes significantly affect female genital health, leading to a constellation of symptoms known as the genitourinary syndrome of menopause (GSM) [1]. Vulvovaginal atrophy (VVA), a core component of GSM, affects approximately half of postmenopausal women and is associated with dryness, irritation, pruritus, dyspareunia, and adverse effects on emotional well-being, body image, and sexuality. Despite its prevalence and impact, VVA remains underreported and underdiagnosed, partly due to stigma and a lack of appropriate assessment tools [2].

Patient-reported outcome measures (PROMs) are central to assessing symptoms that cannot be directly quantified but substantially affect quality of life [3]. These instruments translate subjective experiences into standardised, analytically usable data, thereby facilitating clinical decision-making and the evaluation of therapeutic effectiveness [4]. For research findings to be valid and comparable, PROMs must demonstrate robust psychometric properties and cultural appropriateness [5, 6].

The Day-to-Day Impact of Vaginal Aging questionnaire (DIVA)[7] is a validated instrument that quantifies the impact of genital ageing on quality of life across four domains: daily activities, emotional well-being, sexual function, and self-concept/body image. The questionnaire has demonstrated good psychometric performance and sensitivity to treatment effects [8], and has been culturally adapted into several languages and cultural contexts[9–13]. However, no validated Portuguese version is available.

Adapting the original DIVA questionnaire to the Portuguese DIVAS-Q required more than a literal translation. The original DIVA was developed for postmenopausal women and uses the concept of vaginal ageing. In the present study, the wording was adapted to “vulvovaginal atrophy symptoms” because the target construct was the Day-to-Day impact of symptoms commonly associated with vulvovaginal atrophy, regardless of whether these symptoms occurred exclusively in the context of chronological ageing or menopause.

This broader terminology was considered clinically relevant because vulvovaginal atrophy-like symptoms may also occur in premenopausal women in association with iatrogenic, hormonal, dermatological, postpartum, lactational, oncological, or other gynaecological conditions. The acronym DIVAS-Q (Day-to-Day Impact of Vulvovaginal Atrophy Symptoms Questionnaire) was therefore adopted to identify the European Portuguese-adapted version and to reduce the potentially stigmatising connotations of the term “ageing”, particularly among younger symptomatic women. Nevertheless, this conceptual broadening should be interpreted within the scope of the present validation sample: adult women in Portugal reporting at least one vulvovaginal atrophy-related symptom.

## Methods

### Ethical approval, permission, confidentiality and study registration

This clinical study was conducted in accordance with the current amendments to the International Conference on Harmonisation – Good Clinical Practice (ICH-GCP) guidelines and with the ethical codes applicable to the locations where the study was conducted, including, but not limited to, Portuguese Law No. 21/2014 of April 16 and all decisions and instructions from the relevant authorities. The study was approved by the Ethical Commission of the Local Health Unit of the Leiria Region, Santo André Hospital (ID: 06/2022); the 2022 Ethical Commission for Health of the Regional Health Administration of Lisbon and Tagus Valley (CES ARS-LVT) (ID: 6047/2022); and the Clinical Direction of the Dom Manuel de Aguiar Hospital (authorisation letter signed on 20 January 2022).

Although the Day-to-Day Impact of Vaginal Aging questionnaire is copyrighted [7], it is available free of charge from the authors, and no written permission is required for its use, provided the following conditions are strictly observed: 1 - we refer to the questionnaire by its full name, `Day-to-Day Impact of Vaginal Aging questionnaire`, and include the appropriate citation; 2 - modifications may be made without written permission, provided they are clearly identified.

We obtained participants’ free, informed consent and voluntary collaboration and ensured confidentiality and anonymity, allowing them to withdraw from the study at any time. Any of their personal data will not identify participants. Only the Principal Investigator had access to the list linking identification numbers to participants’ personal data.

This study was prospectively registered on ClinicalTrials.gov (ID: NCT07250490) on 2022-10-02.

### Study design and participants

A multicentre observational validation study was conducted in Portugal from October 2022 to January 2024. Women were recruited from four clinical settings: a public hospital gynaecology department, a private hospital, a private gynaecology practice, and a primary health care unit. Eligible participants were adult women (> 18 years) reporting at least one symptom of vulvovaginal atrophy (e.g., dryness, irritation, pruritus, burning, soreness, dyspareunia, bleeding, or malodorous discharge) that occurred spontaneously or in association with urination or sexual activity. Both pre- and post-menopausal women were included. Exclusion criteria included pregnancy, active gynaecological infection, current oncological treatment, inability to complete questionnaires due to cognitive or physical impairment, and insufficient proficiency in Portuguese.

The sample size for the participants was determined using the rule of thumb of at least 10 participants per scale item [14], corresponding to 23 × 10, which is at least 230 participants.

To increase the reliability of the scale, the ideal procedure is to develop it on sample A, whether cross-sectional or longitudinal, and then to test it on an independent sample B [15]. The scale was developed in California, United States of America, and then tested again in Portugal using four independent samples.

### Translation and cultural adaptation

The translation and cultural adaptation of the DIVA questionnaire [7] followed internationally accepted guidelines from the Consensus-based Standards for the Selection of Health Measurement Instruments (COSMIN) initiative. They included cognitive pre-testing with women experiencing vulvovaginal atrophy symptoms.

The translation and cultural adaptation process included expert review and cognitive debriefing. The expert panel comprised 5 gynaecologists, 2 physiotherapists, 5 nurses specialising in maternal health, and 3 general practitioners: 2 psychologists, 1 methodologist, and 3 bilingual researchers, with clinical or methodological expertise in women’s health, vulvovaginal symptoms, PROM adaptation, and questionnaire validation. After forward translation, reconciliation, and back-translation, the expert panel reviewed semantic, idiomatic, experiential, and conceptual equivalence between the original DIVA and the Portuguese version. Attention was given to the terms “vaginal ageing”, “vulvovaginal symptoms”, sexual activity items, and wording that could be interpreted as stigmatising or difficult to understand.

Cognitive debriefing interviews were then conducted in two rounds, with 10 patients per round in face-to-face discussions. Adult women reporting vulvar or vaginal symptoms were asked whether each item was clear, relevant, acceptable, and easy to answer, and whether any important symptom impact was missing. Participants were also invited to explain selected items in their own words and to identify any ambiguous or uncomfortable wording.

This preliminary cognitive approach for patients attending routine gynaecological appointments helps ensure that respondents understand the questions as intended by the developers and can answer them appropriately, thereby reflecting their experience [16, 17]. Minor wording changes were introduced to improve clarity and cultural appropriateness. No items were removed, and no new items were added, as participants considered the content relevant and sufficiently comprehensive for assessing the day-to-day impact of vulvovaginal atrophy-related symptoms. Controversy was observed in domain 3 (sexual) regarding questions 10, 11, and 16 through 18; some women without sexual activity with a partner preferred a null response to all items in the sexual domain; however, to maintain consistency with the original construct and because this preference among women without sexual activity was not consensual, only a null response was reattained for the original items in this domain. The final wording preserved the original four-domain structure while improving acceptability in the Portuguese context.

### Data collection and instruments

Participants completed a paper-based survey covering sociodemographic and clinical variables, the Portuguese DIVAS-Q (Supplement 1), the Vulvovaginal Symptom Questionnaire (VSQ), a self-developed anchor questionnaire, the European Portuguese version of the EuroQol 5-Dimension (EQ-5D) [18], and the European Portuguese short version 6 - Female Sexual Function Questionnaire (FSFI-6)[19]. A subset of participants completed the DIVAS-Q again after 3–30 days to assess test–retest reliability. Data collection took place from 1st October 2022 to 1st January 2024.

The DIVA [7] assesses and quantifies the impact of female genital ageing. It consists of 23 items organised into four constructs: Factor 1 (F1) - daily activities (D1 to D5); Factor 2 (F2) - emotional well-being (D6 to D9); Factor 3 long (F3-L) - sexual function (D10 to D18); and Factor 3 short (F3-S) - sexual function without recent sexual activity (D10, D11 and D16 to D18); and Factor 4 (F4) - self-concept/body image (D19 to D23). Two versions of the questionnaire are available: the full version with 23 items, which includes four items within the sexual function construct that can be answered only by sexually active women; and a shorter version with 19 items, which can be applied regardless of sexual activity status. Each item is rated on a five-point Likert scale, and the score for each dimension is calculated as the mean of the corresponding items. Higher scores indicate a greater adverse impact of atrophic symptoms.

The Vulvovaginal Symptom Questionnaire (VSQ) is a self-developed “anchor” questionnaire used to select and recruit women with potential vulvovaginal atrophy into our population. It asks women to rate the overall subjective bother associated with vulvar or vaginal symptoms, such as dryness, irritation, pruritus, burning, vaginal pain, soreness, dyspareunia, bleeding, or bad-smelling discharge after sexual intercourse or other sexual activity, using a four-level ordered response scale (1-not at all, 2-a little bit, 3-moderately, and 4-extremely). Total scale scores, calculated by averaging item scores, range from 1 to 4; higher scores indicate greater symptom-related bother. This questionnaire used the inclusion criterion of at least one vulvovaginal symptom complaint to recruit participants, but it is not a validated PROM. We hypothesised that women reporting a greater impact of vaginal symptoms on their activities of daily living, emotional well-being, sexual functioning, and self-concept and body image in the respective DIVA questionnaire scales would also report greater overall bother associated with their vaginal symptoms.

The Portuguese FSFI-6 [19] is a brief, quick-to-complete, valid and reliable questionnaire for assessing female sexual dysfunction (arousal, lubrication, orgasm, and pain) over the past month. Total scores, calculated by averaging individual item scores, range from 1 to 5 for desire and satisfaction, and from 0 to 5 for excitation, lubrication, orgasm, and pain; for the latter, higher values indicate less pain or discomfort. The total score ranges from 2 to 30, with higher scores indicating better sexual function. We hypothesised that women reporting a greater impact of their vaginal symptoms on sexual functioning, as measured by the DIVAS-Q sexual functioning scale, would also report worse overall sexual function. The Portuguese version of EQ-5D [18] provides two essential components of any measure of health-related quality of life: a profile describing health status across domains or dimensions, and a numeric value associated with that profile. It describes health in five dimensions: mobility, personal care, usual activities, pain/malaise, and anxiety/depression. Each dimension has three levels of severity (1 - without, 2 - moderate, and 3 - extreme problem) and may correlate with the fourth dimension of the DIVAS-Q.

### Statistical analysis

Descriptive statistics were computed for all items and scale scores. The psychometric evaluation followed a multidimensional framework comprising: (1) score distributions, including floor and ceiling effects; (2) structural validity assessed via confirmatory factor analysis (CFA); (3) internal consistency; (4) inter-item and corrected item-total correlations; (5) test-retest reliability; (6) convergent and discriminant validity; and (7) known-groups validity. Statistical significance was set at *p* < .05.

Dimensionality was evaluated using an independent-cluster model CFA to assess whether the hypothesised four-factor structure replicated the original model. Because the DIVAS-Q items use five ordered response categories and several items showed skewness and floor effects, CFA models were estimated using the weighted least squares mean- and variance-adjusted (WLSMV/DWLS) estimator, with robust standard errors and mean-adjusted, scaled test statistics. The original four-factor structure was tested separately for the short (19 items; *n* = 291) and long (23 items; *n* = 243) versions. In the short version, the sexual function factor included items D10, D11, and D16-D18; in the long version, it included items D10-D18. The final long-version model also allowed a residual covariance between D17 and D18, consistent with their closely related content.

Model fit was evaluated using multiple goodness-of-fit indices, following conventional cut-off criteria: Comparative Fit Index (CFI) and Tucker–Lewis Index (TLI) values ≥ 0.95 were considered indicative of good fit and ≥ 0.90 of acceptable fit; Root Mean Square Error of Approximation (RMSEA) values < 0.05 indicated good fit and < 0.08 acceptable fit; Standardised Root Mean Square Residual (SRMR) values < 0.05 were considered excellent and < 0.08 acceptable. Parsimony Normed Fit Index (PNFI) > 0.7 indicates a good balance of parsimony. The chi-square test of exact fit and the chi-square/degrees of freedom ratio (χ²/df) were also reported, with values < 2 indicating a good fit and < 3 acceptable fit.

Internal consistency was assessed using Cronbach’s alpha and McDonald’s omega. Cronbach’s alpha assesses the internal consistency of the scale items, i.e., the extent to which the items covary relative to their sum score. Cronbach’s alpha values are interpreted as follows: < 0.60 - poor reliability; 0.61–0.70 - moderate reliability; 0.71–0.80 - good reliability; 0.8–0.9 - very good reliability; ≥ 0.9 - excellent. Furthermore, when evaluating a second-order factor’s convergent validity, group-specific error should be excluded; thus, the average variance extracted for second-order factors should be at least 5. Inter-item and corrected item–total correlations were examined to assess scale homogeneity (coefficients ≥ 0.30 were deemed satisfactory).

Associations between DIVAS-Q domain scores and external measures were examined using Pearson correlation coefficients. Pearson’s r was chosen because the domain scores were approximately continuous and the relationships were assessed for linearity. Convergent and discriminant validity were evaluated through correlations with theoretically related and less closely related constructs, respectively. Absolute values of r were interpreted as negligible (< 0.10), weak (0.10-0.29), moderate (0.30-0.49), strong (0.50-0.69), or very strong ( > = 0.70). Correlations among the DIVAS-Q dimensions and with the VSQ, FSFI-6, and EQ-5D were calculated separately at baseline and, in the retest subsample, at the second assessment.

A priori hypotheses were specified for construct validity. Positive correlations were expected between DIVAS-Q domains and the VSQ symptom-burden score and EQ-5D, as higher values reflect greater symptom burden or health impact. Negative correlations were expected between DIVAS-Q domains and FSFI-6, particularly for the sexual-function domain, as higher DIVAS-Q scores indicate greater symptom impact. In contrast, higher FSFI-6 scores indicate better sexual function.

Test-retest reliability was assessed in the subsample that completed the DIVAS-Q twice. ICCs were estimated using a two-way absolute agreement, single-measure model, and 95% confidence intervals were obtained via nonparametric bootstrap resampling. Known-groups validity was evaluated by comparing women with lower versus higher symptom burden, defined by the median VSQ score. Group differences were summarised using means, standard deviations, Hedges’ g with 95% bootstrap confidence intervals, and two-sided permutation tests. Because the VSQ was a study-specific symptom-burden questionnaire rather than an established, validated PROM, these analyses were interpreted as supportive evidence of known-groups validity.

Statistical analyses were conducted using Jamovi (v2.6.44), IBM SPSS Statistics (v29), and Python to perform the additional bootstrap and permutation-based calculations requested during revision.

## Results

### Descriptive statistics and assessment of normality

A total of 291 women completed the short version of the DIVAS-Q (19 items), and 243 sexually active women completed the long version (23 items). Participants included both pre- and post-menopausal women, reflecting a wide age range and diverse clinical backgrounds. A subset of fifty women from the normative population repeated the self-administered questionnaire at home between 2 and 30 days after their study visits. It was returned to the study staff by post.

The majority (63%) were older than 55 years (range 33 to 83; mean 58 years) and were white (90.4%). The majority (70.6%) were married or living with a male partner; 10.7% were single; 12.5% were divorced; and 6.2% were widowed. Educational level was widely distributed: 24.6% had studied for only 6 years; 32.9% had completed 12 years of compulsory schooling; and the majority, 42.6%, were highly educated. Most (74.4%) were active employees, with nearly half (50.2%) being directors, specialists, or technicians, and the other half being undifferentiated workers, commerce employees, or administrative employees. 22.1% were retired.

Most (72.9%) were menopausal, and 15.8% had not had a sexually active life, of whom 11.2% had not been sexually active for more than ten years. It was observed that 31.6% reported stress urinary incontinence and 25,61% had some level of genital prolapse. Users of lubricants and local hydrants were 33.6% and 33.2%, respectively; a bit more (37.5%) used local hormonal treatment, but just 7.2% used systemic hormonal treatment. The most frequently reported atrophic genital symptom was dryness (73.9%), followed by dyspareunia (67.7%). Dryness was also the most bothersome reported symptom (31.7%).

Pain or malaise was reported by 53.4%; 47% were anxious or depressed; and 16.4% had mobility problems. The incidence of chronic reported problems included sleeping disorders (47.2%), arterial hypertension (28,3%), diabetes (8.9%), and oncological pathologies (9.3%), with breast cancer the most prevalent among those previously diagnosed with cancer (27 subjects, 40.7%). Most patients completed all items of the Portuguese version of the DIVAS-Q at baseline. Missing responses to individual items were very low (< 5%); the exception was the sexual domain, where they ranged from 5% to 10%. Completion of the questionnaire usually required less than 30 min, including the other 3 questionnaires and the demographic questions.

Descriptive statistics for all items are presented in Appendix 1. Item mean scores ranged from 0.43 (item D1) to 1.67 (item D15), and standard deviations ranged from 0.71 to 1.33.

To assess normality, skewness and kurtosis were analysed [20, 21]; skewness values between − 2 and + 2 and kurtosis values between − 7 and + 7 are considered acceptable for most structural equation modelling techniques. Item distributions showed acceptable skewness and kurtosis for structural equation modelling. As expected in community-based samples using Likert-type PROMs, most items showed positive skewness, reflecting a predominance of lower scores. Floor effects were more pronounced in daily activity items, whereas sexual function and self-concept items displayed broader score distributions. On average, this population is not seriously affected by VVA, with a 0-modal value across most items.

Skewness ranged from 0.261 (D15) to 2.023 (D4), indicating highly right-skewed distributions; this positive skewness reflects a few higher scores and many lower ones. Kurtosis values ranged from − 1.180 (D15) to 3.522 (D4), indicating heavy tails and a leptokurtic, peaked distribution with outliers. Items D1 to D5 showed higher positive skewness (> 1.2) and leptokurtic distributions, suggesting limited impact on daily activities, with fewer reporting higher levels.

Shapiro-Wilk test p-values were < 0.001, indicating significant deviations from normality. Together with the ordered response format and the observed floor effects, these distributional features supported the use of WLSMV/DWLS estimation in the CFA models.

### Structural validity of DIVAS- Q

Confirmatory factor analysis supported the original four-factor structure of the Portuguese DIVAS-Q across both versions. A four-factor model was tested separately for the short version (19 items; *n* = 291) and the long version (23 items; *n* = 243) in sexually active women.

For the long version, the final CFA model was estimated using data from *n* = 243 participants, with missing data handled via listwise deletion. Scaled WLSMV/DWLS fit indices indicated good fit: scaled χ²(223) = 280.9, *p* = .005, χ²/df = 1.26, CFI = 0.973, TLI = 0.970, IFI = 0.974, RMSEA = 0.033, 95% CI [0.019, 0.044], SRMR = 0.040, and PNFI = 0.779.

For the short version, the model was estimated using *n* = 291 participants and showed good fit: scaled χ²(146) = 177.9, *p* = .037, χ²/df = 1.22, CFI = 0.981, TLI = 0.978, IFI = 0.981, RMSEA = 0.027, 95% CI [0.007, 0.041], SRMR = 0.035, and PNFI = 0.771.

Table 1 presents the scaled WLSMV/DWLS fit indices for both versions. These findings confirm that the latent structure of the original instrument is preserved in the Portuguese version.

**Table 1:** Confirmatory factor analyses using WLSMV/DWLS estimation to confirm the four-factor structure of both versions of the DIVAS-Q in the analysed population

Model	*n*	Scaled χ²(df)	χ²/df	CFI	TLI	IFI	RMSEA [95% CI]	SRMR	PNFI
Short version	291	177.9(146)	1.22	0.981	0.978	0.981	0.027 [0.007-0.041]	0.035	0.771
Long version	243	280.9(223)	1.26	0.973	0.970	0.974	0.033 [0.019-0.0. 44]	0.040	0.779

Interpretation of scaled WLSMV/DWLS fit indices and CFA model specification: CFI, TLI, and IFI values >= .95 indicate good fit; RMSEA < .05 and SRMR < .05 indicate good fit/excellent residual fit

### Measurement model

All standardised factor loadings were statistically significant (*p* < .001). In the long version, loadings ranged from 0.670 to 0.948, and in the short version from 0.620 to 0.934, indicating that all items were strong indicators of their respective latent constructs. These results support the adequacy of the measurement model and provide evidence of satisfactory convergent validity across domains (see Appendix 2 and 3).

### Structural model (path coefficients)

**Fig. 1:**
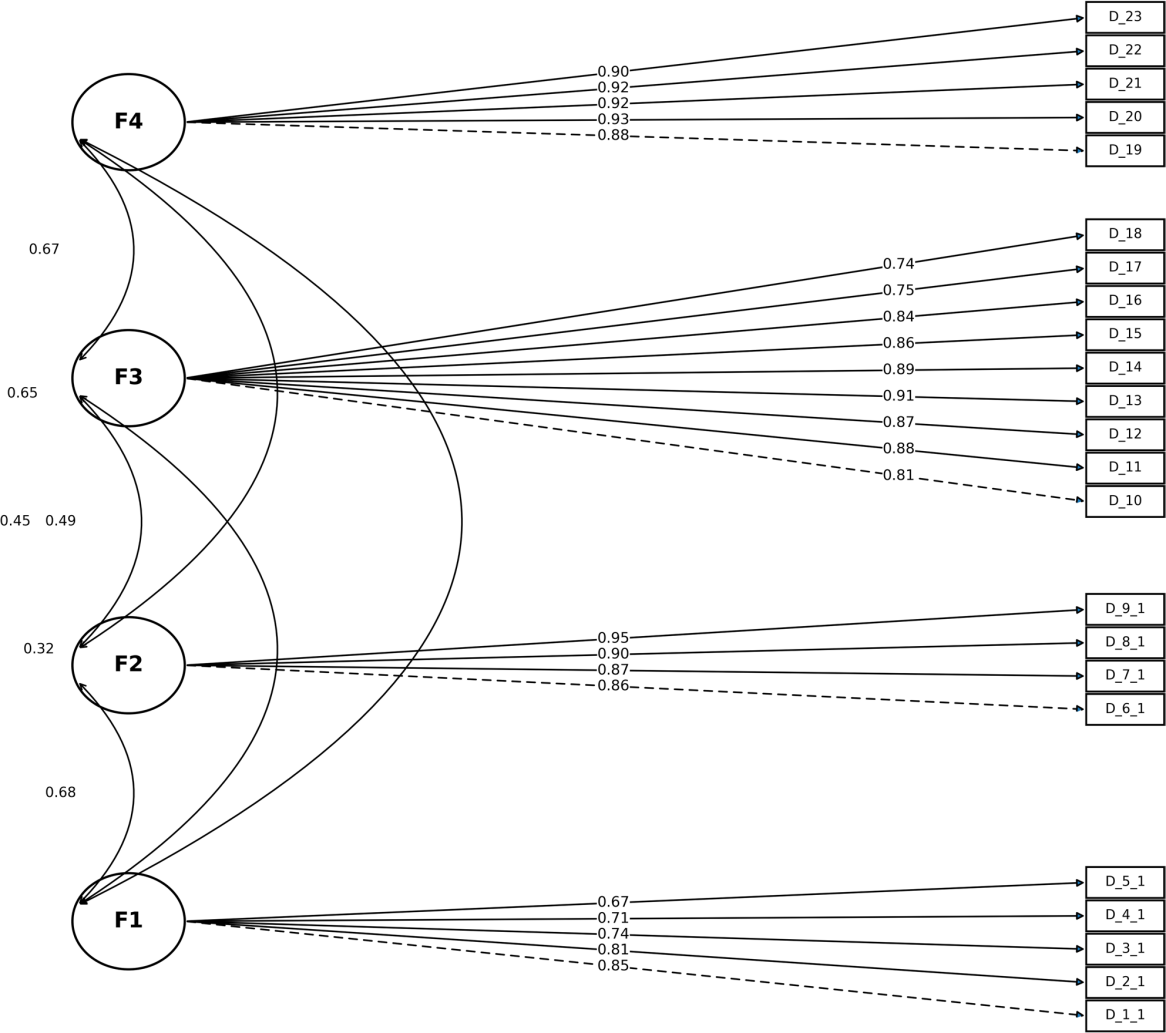
Path diagram DIVAS-Q

**Fig. 2:**
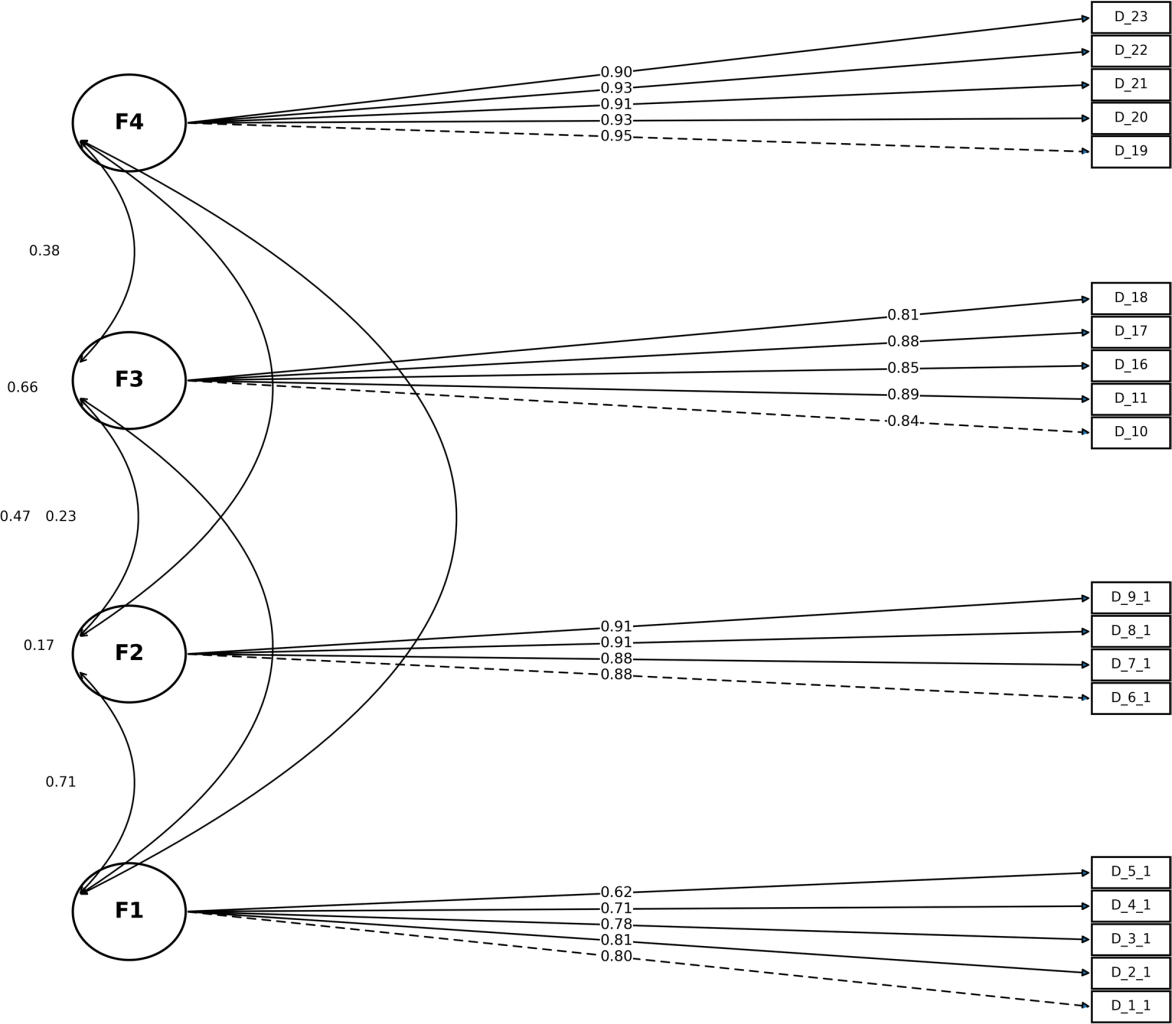
Path diagram DIVAS-Q, short version

Latent correlations between domains were moderate to strong and consistent with the instrument’s theoretical framework, supporting a coherent underlying construct of the day-to-day impact of vaginal ageing. The strongest association was observed between daily activities and emotional well-being (DIVAS-Q long/short), with coefficients of 0.67/0.7, respectively. Emotional well-being also showed robust correlations with self-concept/body image (DIVAS-Q long/short), 0.65/0.66, respectively, and with sexual function (DIVAS-Q long/short), 0.64/0.58, respectively. In contrast, correlations between daily activities and sexual function were weaker (DIVAS-Q long/short: 0.31/0.29, respectively), indicating that these domains capture related but distinct dimensions of vulvovaginal atrophy. The Path Diagram visually depicts the relationships among the factors (Figs. 1 and 2).

### Reliability

Internal consistency was excellent across all DIVAS-Q domains. Cronbach’s alpha coefficients ranged from 0.856 to 0.960, and McDonald’s omega values corroborated these findings. Average variance extracted values exceeded the recommended threshold of 0.50 for all constructs, supporting adequate reliability and convergent validity (Table 2).

**Table 2:** Reliability of the DIVAS-Q Short Version **(Sv)** and Long version (Lv)

Variable		α*	ω1**	ω2**	ω3**	AVE***
F1	Sv	0.856	0.857	0.857	0.855	0.550
	Lv	0.867	0.869	0.869	0.866	0.573
F2	Sv	0.938	0.938	0.938	0.937	0.791
	Lv	0.942	0.942	0.942	0.939	0.802
F3	Sv	0.929	0.930	0.930	0.933	0.728
	Lv	0.956	0.949	0.949	0.949	0.706
F4	Sv	0.957	0.957	0.957	0.957	0.818
	Lv	0.960	0.960	0.960	0.959	0.829

* Cronbach’s alpha coefficients

** McDonald’s omega values

*** Average Variance Extracted

### Inter-item correlations

Inter-item correlations assess the extent to which scores on one item are related to scores on all other items on the scale (see Table 3); they also indicate the extent to which items on a scale assess the same content. Corrected item-total correlations ranged from 0.597 (D5) to 0.916 (D22), and removing any single item did not improve reliability, supporting the retention of all items. These correlations were calculated within each DIVAS-Q domain/subscale rather than across the total multidimensional instrument. This approach was selected because the DIVAS-Q is organised into theoretically distinct domains, and a global item-total correlation across all items could obscure domain-specific item performance. Cronbach’s alpha-if-item-deleted values were also examined within the corresponding domain.

**Table 3:** Item-total correlations

Item (how often your vaginal symptoms- dryness, irritation, soreness or itching, make you feel or interfere with you in the past four weeks)	Corrected Item-Total correlation	Cronbach’s alpha if Item deleted	Correlation
D1. Walk at your usual speed	0.680	0.823	Good
D2. the clothing or underwear you want	0.733	0.813	Good
D3. Use the toilet or wipe yourself after using the toilet	0.696	0.820	Good
D4. Sit for more than an hour	0,687	0.824	Good
D5. Get a good night’s sleep	0.597	0.849	Moderate
D6. Depressed or down	0.812	0.933	Excellent
D7. Embarrassed	0.881	0.911	Excellent
D8. Depressed or resentful	0.870	0.914	Excellent
D9. Bad about yourself	0.853	0.920	Excellent
D10. Your desire or interest in having intercourse or other sexual activity	0,803	0,962	Excellent
D11. How frequently did you have sexual intercourse or other sexual activity	0.847	0.960	Excellent
D12. Your ability to become aroused by sexual activity	0.871	0.959	Excellent
D13. Your ability to be spontaneous about sexual activity	0.892	0.958	Excellent
D14. Your ability to relax and enjoy sexual activity	0.903	0.957	Excellent
D15. The amount of pleasure you experienced during sexual activity	0.887	0.958	Excellent
D16. Your desire or interest in being in a sexual relationship	0.877	0.958	Excellent
D17. Your confidence that you could sexually satisfy a partner	0.775	0.963	Good
D18. Your overall satisfaction with your sexual life	0.775	0.963	Good
D19. My vaginal symptoms make me feel that I am getting old	0.816	0.958	Excellent
D20. I feel undesirable because of my vaginal symptoms	0.885	0.946	Excellent
D21. When I think about my vaginal symptoms, I feel like I have lost something	0.909	0.942	Excellent
D22. My vaginal symptoms make me feel like my body is deteriorating	0.916	0.941	Excellent
D23. I feel less sexy because of my vaginal symptoms	0,875	0,948	Excellent

### Correlations - convergent and discriminant validity

As hypothesised, DIVAS-Q domain scores demonstrated moderate-to-strong correlations with the VSQ and moderate correlations with EQ-5D scores, supporting convergent validity. Since higher values on these scales indicate worse outcomes, these positive correlations indicate that worse DIVAS-Q scores are associated with symptom severity and lower quality of life. At the same time, the DIVAS-Q remains more specific than a generic quality-of-life questionnaire (see Table 4).

Associations with the FSFI-6 were weaker and domain-specific, supporting the conceptual distinction between perceived symptom impact and sexual function. The FSFI-6 applies fully to women with recent sexual activity; therefore, missing or restricted data among women who are sexually inactive or avoid intercourse because of vulvovaginal symptoms may reduce variability and attenuate correlations with DIVAS-Q sexual-function scores. The stronger negative association between F4 (self-concept/body image) and the FSFI-6 suggests that women reporting greater body-image impact also report poorer sexual function, which is theoretically consistent with the FSFI-6’s focus on experienced sexual response.

**Table 4:** Scales correlations at T1 (basal application of DIVAS-Q)

Questionnaires	F1		F2		F3-S		F3		F4	
	*r*	*p*	*r*	*p*	*r*	*p*	*r*	*p*	*r*	*p*
VSQ	0.446	< 0.001	0.614	< 0.001	0.507	< 0.001	0.507	< 0.001	0.578	< 0.001
EQ-5D	0.355	< 0.001	0.313	< 0.001	0.140	< 0.05	0.142	< 0.05	0.338	< 0.001
FSFI-6	-0.064	0.289	-0.082	0.171	0.095	0.113	0.092	0.124	− 0.281	< 0.01

See comprehensive analysis in Appendix 4: Inter-scales Pearson Correlation coefficients

### Test-retest reliability

Test-retest reliability was assessed in a subsample of 50 women who completed the questionnaire twice within a 2-30-day interval, with a median interval of 16.0 days (M = 16.5, SD = 4.14). Using a two-way absolute-agreement single-measure ICC model, temporal stability was excellent across domains: F1 ICC = 0.982, 95% CI [0.959, 0.991]; F2 ICC = 0.984, 95% CI [0.972, 0.993]; F3-S ICC = 0.994, 95% CI [0.989, 0.997]; F3-L ICC = 0.989, 95% CI [0.970, 0.997]; and F4 ICC = 0.986, 95% CI [0.973, 0.995]. These results indicate strong short-term temporal stability, although the very high ICCs should be interpreted in light of the relatively short retest interval and the possibility of recall effects.

### Known-groups validity

Known-groups validity was assessed using the VSQ as a study-specific symptom-burden anchor. Participants were classified into lower- and higher-symptom-burden groups based on the median VSQ score (lower ≤ 1.714; higher > 1.714). As expected, women with higher VSQ symptom burden reported significantly higher DIVAS-Q scores across all domains, with large effect sizes (Table 5). These findings provide supportive evidence that the DIVAS-Q discriminates between women with lower and higher perceived vulvovaginal symptom burden.

We formulated initial hypotheses regarding differences between age groups (see Table 6 for the age-group distribution). Given that menopause typically occurs between 45 and 55 years, we predicted that women older than 55 would have lower DIVAS-Q scores. We used independent-samples t-tests to compare the groups across study variables. Results revealed significant age-related differences in FSFI-6 and EQ-5D scores, but most DIVAS-Q domains showed no significant variation by age group. These findings indicate that the perceived impact of vulvovaginal atrophy symptoms is not solely determined by age.

**Table 5:** Known-groups validity according to VSQ symptom burden

DIVAS-Q domain	Lower VSQ *n*	Lower VSQ M (SD)	Higher VSQ *n*	Higher VSQ M (SD)	Hedges’ g [95% CI]
F1 Daily activities	152	0.27 (0.46)	139	0.74 (0.76)	0.75 [0.52, 0.98]
F2 Emotional well-being	152	0.41 (0.66)	138	1.23 (0.97)	1.00 [0.76, 1.26]
F3-S Sexual function short	133	1.06 (0.94)	130	2.04 (1.03)	0.99 [0.75, 1.27]
F3-L Sexual function long	133	1.03 (0.94)	131	2.02 (1.07)	0.99 [0.73, 1.27]
F4 Self-concept/body image	152	0.66 (0.81)	139	1.69 (1.12)	1.05 [0.79, 1.34]

Interpretation of symptom-burden groups using the median VSQ score: VSQ = Vulvovaginal Symptom Questionnaire. Lower symptom burden was defined as VSQ < = 1.714 and higher symptom burden as VSQ > 1.714. All two-sided permutation tests were *p* < .001

**Table 6:** Differences according to women’s age ≤ 55 versus > 55 years

Variable	Levene’s Significance	*p*-value (2-tailed)
F1	0.058	0.051
F2	0.754	0.438
F3-S	0.189	0.921
F3	,148	0.648
F4	0.011	0.120
FSFI- 6	0.416	< 0.001
VSQ	0.928	0.614
EQ_5D	> 0,001	0.005

## Discussion

As science advances and novel research questions emerge, new scales are needed. Scale development is not, however, an obvious or straightforward endeavour [22, 23]. This study translated, culturally adapted, and validated the DIVAS-Q questionnaire into European Portuguese, providing a tool to assess the vulvovaginal atrophy.

A key contribution of this study was the inclusion of both pre- and post-menopausal symptomatic women. While vulvovaginal atrophy is traditionally associated with menopause, similar symptoms may also occur in younger women due to hormonal contraception, lactation, oncological treatments, dermatological conditions, or other gynaecological contexts. The additional known-groups analysis based on VSQ symptom burden showed large differences across all DIVAS-Q domains, whereas age-based differences were smaller and less consistent. This pattern suggests that the perceived day-to-day impact captured by the DIVAS-Q is more closely aligned with symptom burden than with chronological age alone. However, because the VSQ was study-specific, these findings should be interpreted as supportive rather than definitive criterion-based evidence.

During cognitive debriefing, several participants appeared to use the terms vulva and vagina interchangeably. Therefore, in this version, we assume that the complaints are in the vulva and the vaginal area (female external genitals). However, in each sentence of the questionnaire, we refer to “vagina” to simplify understanding for women (Supplement A – Portuguese version of DIVAS–Q). This adaptation ensures clarity while maintaining the original psychometric properties across the four domains of activities of daily living, emotional well-being, sexual functioning, and self-concept and body image. Consequently, this instrument provides clinicians and researchers with a culturally appropriate tool to assess the burden of vaginal atrophy symptoms in Portuguese-speaking populations, facilitating more targeted and effective symptom evaluation and treatment interventions.

This study provides robust evidence supporting the validity and reliability of the Portuguese DIVAS-Q as a patient-reported outcome measure for assessing the multidimensional impact of vulvovaginal atrophy.

The original four-factor structure of the DIVA questionnaire was successfully replicated in both the long and short versions, and all psychometric properties met or exceeded recommended standards. These validation results are consistent with all prior validation results reported7-13.

Internal consistency and test–retest reliability were excellent across all domains, confirming the instrument’s stability and precision. Convergent and discriminant validity analyses showed the expected patterns of association with related and unrelated measures, thereby supporting the construct validity of the DIVAS-Q.

Items D14, “Your ability to relax and enjoy sexual activity”, and D15, “The amount of pleasure you experienced during sexual activity”, had the highest mean scores and the lowest skewness, indicating greater overall impact and suggesting they may be key indicators of disease burden and contributors to the factor structure. By contrast, D1, “Walk at your usual speed”, and D4, “Sit for more than an hour”, had low mean scores and high skewness, indicating limited perceived impact in this sample and suggesting reduced discriminatory capacity.

The strongest latent associations were observed between emotional well-being and self-concept/body image, underscoring the substantial psychological and psychosocial burden associated with vulvovaginal atrophy symptoms. These findings reinforce the importance of comprehensive assessment tools that extend beyond physical symptoms to capture emotional and quality-of-life dimensions.

Very high correlations between the short and long sexual function versions (0.98–0.99) indicate that both formats capture essentially the same construct of sexual impact in women with and without recent sexual activity.

The conceptual reasons for the weak correlation between DIVAS-Q F3 and FSFI-6 may reflect that the DIVAS-Q sexual dimension assesses the impact of vaginal atrophy on sexual life (limitations, discomfort, distress). In contrast, the FSFI-6 focuses on specific sexual function domains (desire, arousal, lubrication, orgasm, satisfaction, pain) over the past 4 weeks. A woman may report substantial perceived impact of vaginal atrophy on sexuality (high DIVAS-Q sexual scores) yet maintain relatively preserved sexual function on the FSFI-6 (e.g., due to treatment, partner adaptation, or reduced frequency), leading to a mismatch between impact and function scores.

The high consistency observed over time supports both the scale’s reliability and the study’s non-interventional design. Nevertheless, the emotional well-being domain was the least stable over time, whereas the sexual activity domain was the most stable in this population. These findings are consistent with the well-recognised variability of emotional states, which may be influenced by multiple factors, including physical aspects such as genital ageing or atrophy, as well as psychological, social, and economic factors. By contrast, greater stability in sexual activity within this age group is generally consistent with current understanding and reflects the high rates of stable partnerships.

The process of linguistic translation is a complex challenge, particularly when translating from an Anglo-Saxon language to a Romance language. However, this process extends beyond this specific case to other cultures and languages regarding the validation of health measures that significantly impact quality of life; we therefore consider this work an important health milestone for women in Portugal.

Some limitations should be acknowledged. The overall symptom burden in the sample was relatively low, and most participants were Caucasian, which may limit generalisability. Although the CFA was estimated using WLSMV/DWLS, which is appropriate for ordered categorical indicators and floor effects, the measurement structure should be replicated in larger and more diverse samples. Known-groups validity was strengthened by the VSQ symptom-burden comparison. However, this anchor was study-specific and should be complemented in future studies by external clinical criteria such as menopausal status, dyspareunia, hormonal therapy, clinician-rated severity, or confirmed diagnoses. Responsiveness to change and minimal clinically important differences were not assessed and should be addressed in future longitudinal and interventional studies.

Further longitudinal and intervention studies are needed before the instrument can be recommended for monitoring treatment response or for interpreting clinically meaningful change over time.

## Conclusion

The European Portuguese DIVAS-Q demonstrated good psychometric properties for assessing the day-to-day impact of vulvovaginal atrophy-related symptoms among symptomatic women in Portugal. The present validation evidence supports its use across reproductive stages and clinical settings. However, its application in other Portuguese-speaking countries requires additional linguistic, cultural, and psychometric validation before cross-national generalisation can be assumed.

Future studies should further examine measurement invariance, responsiveness to change, and minimal clinically important differences, so that the DIVAS-Q can be used with greater confidence to evaluate symptom trajectories and treatment-related change.

## Appendices

### Appendix 1. Descriptive statistics of DIVAS-Q

**Figure:**
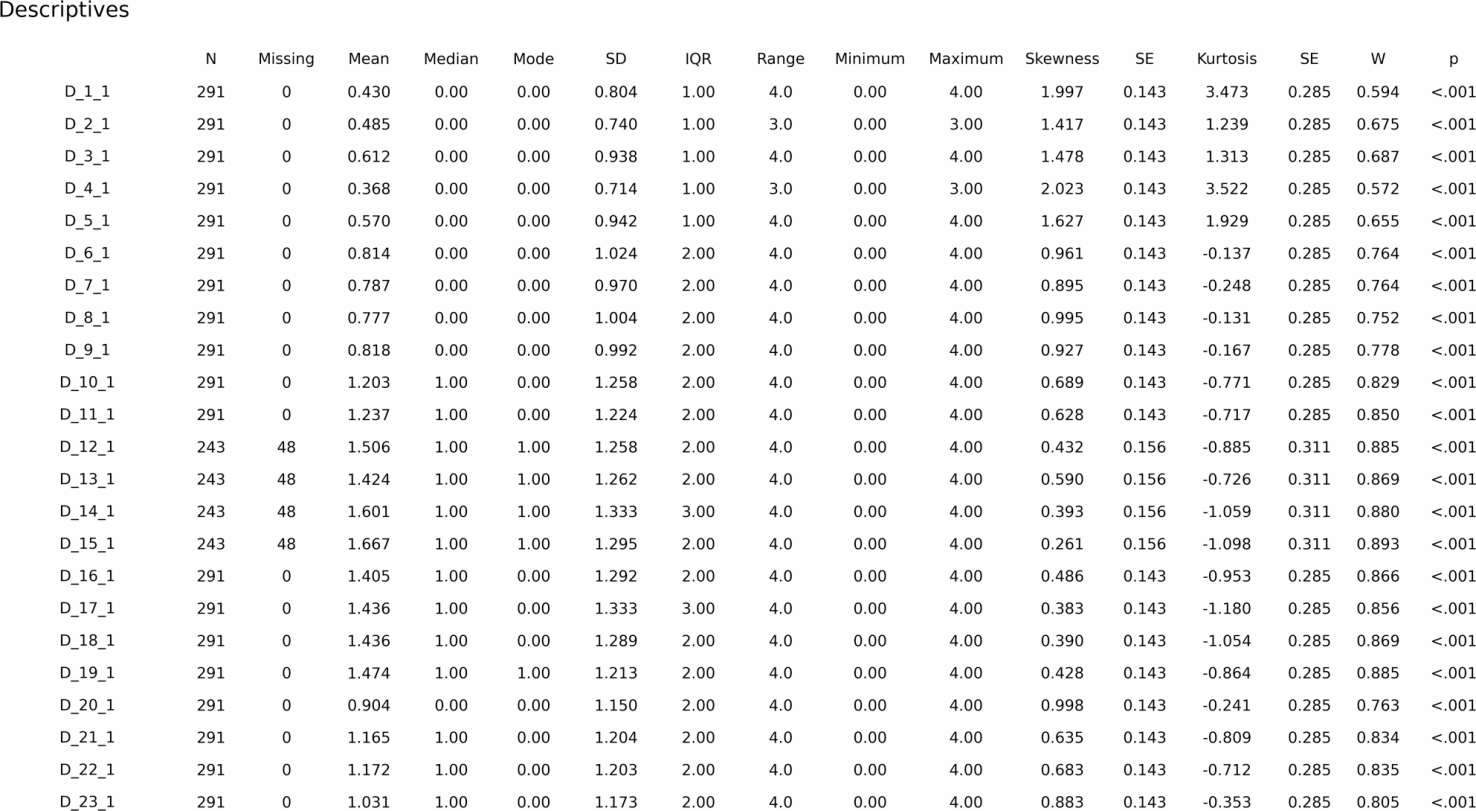

(N) Number.

(SD) Standard Deviation.

(IQR) Interquartile Range.

(SE) Standard Error.

### Appendix 2 – Measurement model reliability of DIVAS-Q

**Figure:**
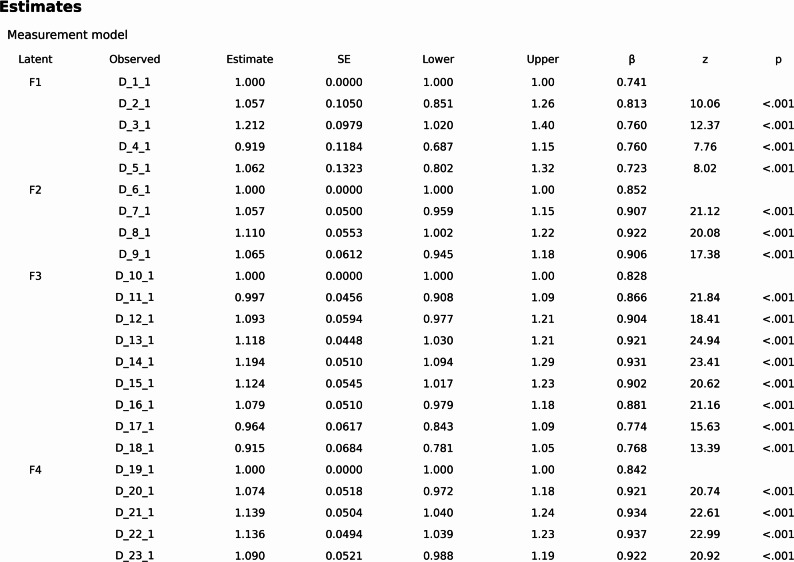

(SE) Standard Error.

(β) standardised path coefficients.

### Appendix 3: Measurement model reliability of DIVAS-Q, short version

**Figure:**
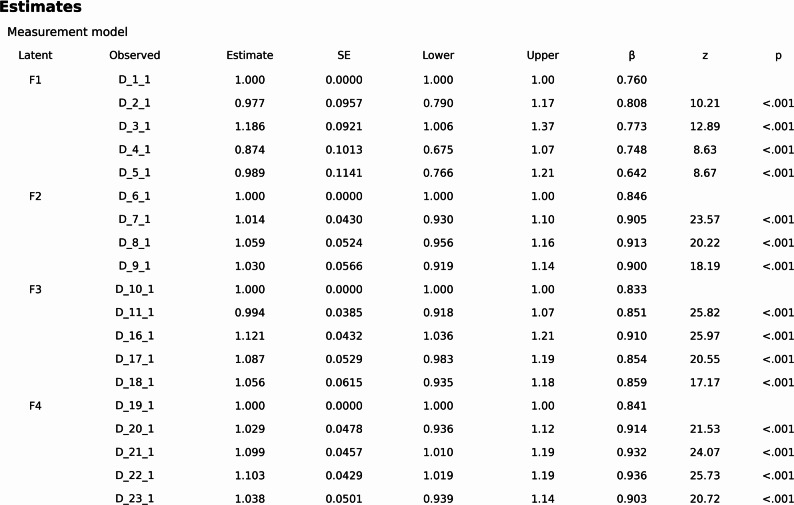

### Appendix 4: Inte- scales pearson correlation coefficients

**Figure:**
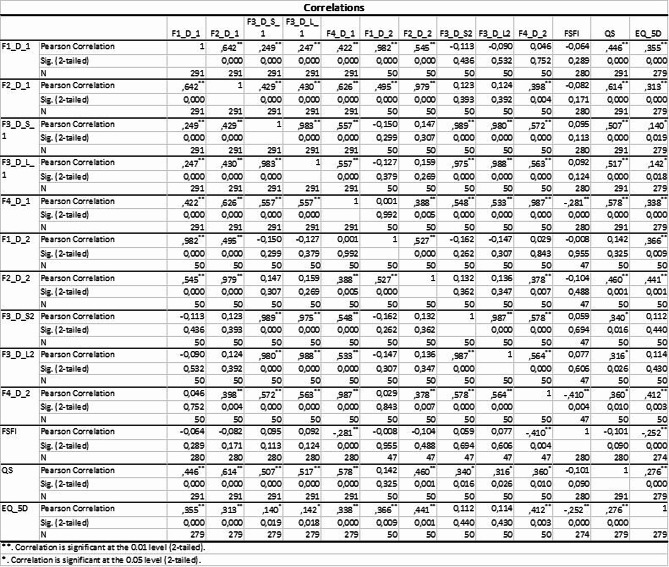

(D1) Time 1 of application of the 1st DIVAS-Q.

(D2) Time 2 of the application of a subset of 50 DIVAS-Q retest.

(FSFI) 6 Items - Female Sexual Function Index.

(QS) Vaginal Symptoms Questionnaire.

(EQ_5D) Euro Qol 5 D.

## Supplementary Information

Below is the link to the electronic supplementary material.

Supplementary Material 1

## Data Availability

Individual questionnaires are not publicly available due to privacy and ethical restrictions. Datasets on which the conclusions of the manuscripts rely are publicly available at datarepositorium.uminho.pt: 10.34622/datarepositorium/TGPO9C.
