# Supplementary material for: Validation of the DIVAS-Q (Day-to-Day Impact of Vulvovaginal Atrophic Symptoms Questionnaire): a Portuguese patient-reported outcome measure
Source: J Patient Rep Outcomes. 2026 Aug 14;10:147. doi: 10.1186/s41687-026-01172-4 (PMC13486427; doi:10.1186/s41687-026-01172-4)
Supplement: Supplementary file 1 [file 41687_2026_1172_MOESM1_ESM.docx]

Supplement 1 - Portuguese version of DIVAS – Q

**IMPACTO DIÁRIO DOS SINTOMAS DE ATROFIA VULVOVAGINAL – QUESTIONÁRIO (DIVAS – Q)**

Estamos interessados em entender o impacto dos sintomas vulvovaginais (mas que se designarão somente como vaginais para facilitar entendimento corrente) como: secura vaginal, dor, irritação e comichão no seu dia-a-dia. Para cada pergunta abaixo, marque a resposta que melhor descreve como as suas atividades, relacionamentos e sentimentos foram afetados por qualquer um destes sintomas durante as últimas 4 semanas.

**Parte A. Durante as últimas 4 semanas, quanto é que os sintomas vaginais como: secura, ardor, irritação ou comichão, foram desconfortáveis ou interferiram na sua capacidade de:**

1. Andar à velocidade habitual?

□0 Nada	□1 Um pouco	□2 Moderadamente	□3 Bastante	□4 Extremamente

2. Usar a roupa e lingerie que quer?

□0 Nada	□1 Um pouco	□2 Moderadamente	□3 Bastante	□4 Extremamente

3. Usar a casa de banho ou limpar-se após o uso desta?

□0 Nada	□1 Um pouco	□2 Moderadamente	□3 Bastante	□4 Extremamente

4. Ficar sentada mais de 1 hora?

□0 Nada	□1 Um pouco	□2 Moderadamente	□3 Bastante	□4 Extremamente

5. Ter uma boa noite de sono?

□0 Nada	□1 Um pouco	□2 Moderadamente	□3 Bastante	□4 Extremamente

**Parte B. Durante as últimas 4 semanas, com que frequência os sintomas vaginais como: secura, ardor, irritação ou comichão, fizeram com que se sentisse:**

6. Deprimida ou em baixo?

□0 Nunca	□1 Raramente	□2 Às vezes	□3 Frequentemente	□4 Quase sempre

7. Embaraçada?

□0 Nunca	□1 Raramente	□2 Às vezes	□3 Frequentemente	□4 Quase sempre

8. Frustrada ou ressentida?

□0 Nunca	□1 Raramente	□2 Às vezes	□3 Frequentemente	□4 Quase sempre

9. Mal consigo própria?

□0 Nunca	□1 Raramente	□2 Às vezes	□3 Frequentemente	□4 Quase sempre

**Parte C. As perguntas que se seguem são sobre o impacto dos seus sintomas, nas relações sexuais e outros tipos de atividade sexual, como autoestimulação ou masturbação. Durante as últimas 4 semanas, sintomas vaginais como: secura, ardor, irritação ou comichão, afetaram:**

10. O seu desejo ou interesse em ter relações sexuais ou outros tipos de atividade sexual (autoestimulação ou masturbação)?

□0 Nada	□1 Um pouco	□2 Moderadamente	□3 Bastante	□4 Extremamente

11. A frequência com que teve relações sexuais, ou outros tipos de atividade sexual (incluindo autoestimulação ou masturbação)?

□0 Nada	□1 Um pouco	□2 Moderadamente	□3 Bastante	□4 Extremamente

12. A sua capacidade de ficar excitado durante a atividade sexual (incluindo autoestimulação ou masturbação)?

□0 Nada	□1 Um pouco	□2 Moderadamente	□3 Bastante	□4 Extremamente

□ Não aplicável- não tive atividade sexual de nenhum tipo recentemente.

13. A sua capacidade de espontaneamente se dedicar á atividade sexual (incluindo autoestimulação e masturbação)?

□0 Nada	□1 Um pouco	□2 Moderadamente	□3 Bastante	□4 Extremamente

□ Não aplicável- não tive atividade sexual de nenhum tipo recentemente.

14. A sua capacidade de relaxar e desfrutar da atividade sexual (incluindo autoestimulação ou masturbação)?

□0 Nada	□1 Um pouco	□2 Moderadamente	□3 Bastante	□4 Extremamente

□ Não aplicável- não tive atividade sexual de nenhum tipo recentemente.

15. O nível de prazer que experienciou durante a atividade sexual (incluindo autoestimulação ou masturbação)?

□0 Nada	□1 Um pouco	□2 Moderadamente	□3 Bastante	□4 Extremamente

□ Não aplicável- não tive atividade sexual de nenhum tipo recentemente.

16. O seu desejo ou interesse em ter um relacionamento sexual?

□0 Nada	□1 Um pouco	□2 Moderadamente	□3 Bastante	□4 Extremamente

17. A sua confiança em ser capaz de satisfazer sexualmente um parceiro(a)?

□0 Nada	□1 Um pouco	□2 Moderadamente	□3 Bastante	□4 Extremamente

18. A sua satisfação geral com a sua vida sexual?

□0 Nada	□1 Um pouco	□2 Moderadamente	□3 Bastante	□4 Extremamente

**Parte D. As afirmações que se seguem descrevem a forma pela qual os seus sintomas vaginais podem ter afetado o que sente por si e pelo seu corpo. Por favor, indique o quão verdadeira cada uma das seguintes afirmações foi para si durante as últimas 4 semanas.**

19. Os meus sintomas vaginais fazem sentir-me como se estivesse a envelhecer.

□0 Nada	□1 Um pouco	□2 Às vezes	□3 Muitas vezes	□4 Sempre

20. Sinto-me indesejável devido aos meus sintomas vaginais.

□0 Nada	□1 Um pouco	□2 Às vezes	□3 Muitas vezes	□4 Sempre

21. Quando penso nos meus sintomas vaginais, sinto como se tivesse perdido algo.

□0 Nada	□1 Um pouco	□2 Às vezes	□3 Muitas vezes	□4 Sempre

22. Os meus sintomas vaginais fazem-me sentir como se o meu corpo se estivesse a deteriorar

□0 Nada	□1 Um pouco	□2 Às vezes	□3 Muitas vezes	□4 Sempre

23. Sinto-me menos sexualmente atraente (sexy) devido aos meus sintomas vaginais.

□0 Nada	□1 Um pouco	□2 Às vezes	□3 Muitas vezes	□4 Sempre

Agradecemos as suas respostas........................................................................................................................

**End of DIVAS questionnaire**

**Acknowledgments for Authors**

Adenda a DIVAS -Q

A escala é composta por duas versões:

Versão curta – composta por 19 Itens, pode ser respondida por todas as mulheres independentemente de terem ou não atividade sexual.

Versão longa – composta por 23 Itens, com 4 itens adicionais no domínio da função sexual pode ser respondida por mulheres com atividade sexual nas últimas 4 semanas.

A escala é composta por 4 domínios:

Domínio das atividades da Vida diária: itens 1 a 5

Domínio do bem-estar emocional: itens 6 a 9

Domínio da função sexual – versão curta: itens 10, 11 e 16-18

Domínio da função sexual- versão longa: itens 10 a 18

Domínio do autoconceito e autoimagem: itens 19 a 23

Pontuação recomendada:

As pontuações totais para cada domínio da escala são calculadas pela média das pontuações para os itens individuais correspondentes. O intervalo para todos os domínios da escala é de 0 a 4, com pontuações mais altas denotando maior impacto dos sintomas vulvovaginais.

Permissões:

Embora o DIVAS – Q seja protegido por direitos de autor, ele está disponível gratuitamente pelos autores e nenhuma permissão por escrito é necessária para sua utilização.

Poderão ser efetuadas modificações a esta versão do questionário sem permissão por escrito, contudo deverá ser remetido ao autor responsável por esta versão a publicação dessas alterações.

Esta versão do questionário DIVAS – Q resulta da validação psicométrica da versão original cuja fonte bibliográfica se indica:

Huang AJ, Gregorich SE, Kuppermann M, et al. Day-to-Day Impact of Vaginal Aging questionnaire: A multidimensional measure of the impact of vaginal symptoms on functioning and well-being in postmenopausal women. *Menopause*. 2015;22(2):144-154. doi:10.1097/GME.0000000000000281
